# Infodemiology and Infoveillance: Framework for an Emerging Set of Public Health Informatics Methods to Analyze Search, Communication and Publication Behavior on the Internet

**DOI:** 10.2196/jmir.1157

**Published:** 2009-03-27

**Authors:** Gunther Eysenbach

**Affiliations:** ^1^Centre for Global eHealth InnovationUniversity Health NetworkTorontoCanada; ^2^Department of Health Policy, Management, and EvaluationUniversity of TorontoTorontoCanada

**Keywords:** epidemiology, Internet, forecasting, population surveillance, influenza, human, consumer health information, epidemiological indicators, quality indicators, information storage and retrieval, biosurveillance, syndromic surveillance

## Abstract

Infodemiology can be defined as the science of distribution and determinants of information in an electronic medium, specifically the Internet, or in a population, with the ultimate aim to inform public health and public policy. Infodemiology data can be collected and analyzed in near real time. Examples for infodemiology applications include: the analysis of queries from Internet search engines to predict disease outbreaks (eg. influenza); monitoring peoples' status updates on microblogs such as Twitter for syndromic surveillance; detecting and quantifying disparities in health information availability; identifying and monitoring of public health relevant publications on the Internet (eg. anti-vaccination sites, but also news articles or expert-curated outbreak reports); automated tools to measure information diffusion and knowledge translation, and tracking the effectiveness of health marketing campaigns. Moreover, analyzing how people search and navigate the Internet for health-related information, as well as how they communicate and share this information, can provide valuable insights into health-related behavior of populations. Seven years after the infodemiology concept was first introduced, this paper revisits the emerging fields of infodemiology and infoveillance and proposes an expanded framework, introducing some basic metrics such as information prevalence, concept occurrence ratios, and information incidence. The framework distinguishes supply-based applications (analyzing what is being published on the Internet, eg. on Web sites, newsgroups, blogs, microblogs and social media) from demand-based methods (search and navigation behavior), and further distinguishes passive from active infoveillance methods. Infodemiology metrics follow population health relevant events or predict them. Thus, these metrics and methods are potentially useful for public health practice and research, and should be further developed and standardized.

## Classical and Recent Infodemiology Studies

Gunther Eysenbach The Internet has made measurable what was previously immeasurable: The distribution of health information in a population, tracking (in real time) health information trends over time, and identifying gaps between information supply and demand. [1]

Galileo Galilei Count what is countable, measure what is measurable. What is not measurable, make measurable.

A few weeks ago, a paper published in the journal Nature by scientists associated with Google made worldwide headlines: Ginsberg and colleagues discussed how monitoring search queries on Google can be used to predict influenza outbreaks in the United States [2]. Data from this study was used to develop the Google Flutrends application. What was frequently missed in lay media reports was the fact that this was not an entirely novel idea. In fact, exactly the same methods have been employed and evaluated at the Centre for Global eHealth Innovation since 2002, under the label “infodemiology”. An award-winning paper published in 2006 by Eysenbach was the first to show a correlation between influenza-related searches on Google and influenza cases occurring in the following week in Canada [1]. As Google did not share search data with external researchers, a “trick” was used to obtain these data: A keyword-triggered ad on Google was purchased, allowing access to statistics reflecting search and click behaviour of Google users. This pioneering study also showed that Internet searches preceded doctors’ visits to sentinel physicians by 1 week (a fact which was later also confirmed by the Ginsberg study), pointing to the circumstance that often people first consult the Internet before going to a doctor. As early as 2003, a similar methodology was employed to evaluate whether search behavior changed before the SARS outbreak; however, at that time “in our search term experiment it did not seem to be sensitive enough [to detect] SARS” [3]. These early studies have inspired and motivated others to explore correlations between search behavior, information on the Internet, and public health relevant events. Wilson and Brownstein published a paper suggesting that chatter on the Internet preceded official announcement of a Listeriosis outbreak [4]. A number of other studies have replicated findings from the Eysenbach study on the relationship between Internet search behavior and influenza incidence [2,5,6]. A seminal paper published by Cooper and colleagues in this journal explored the relationship between search behavior for cancer (information demand), cancer incidence, cancer mortality, and news coverage (information supply) [7].

These studies are part of a growing body of literature that has been called “infodemiology” or, if the primary aim is surveillance, “infoveillance” [8] studies—automated and continuous analysis of unstructured, free text information available on the Internet. This includes analysis of search engine queries (the “demand” side), but also what is being published on websites, blogs, etc (the “supply” side).

Seven years after the concept was first introduced [9], this paper revisits the emerging fields of infodemiology and infoveillance and proposes an expanded framework. This paper also aims to illustrate the potential by suggesting applications for syndromic surveillance and management of public health emergencies, quality monitoring of information on the Internet, knowledge translation, health communication, health marketing, and populomics, including collecting behavioral measures at a population level for public health policy and practice. To illustrate the potential applications further and to form a crystallization point for collaborations we are working on the Infovigil project at the Centre for Global eHealth Innovation, which is a system allowing researchers, public health professionals, and the public to collect and monitor some of the metrics described below.

Whether infodemiology indicators follow public health relevant events or predict them, the main thesis of this paper is that infodemiology metrics and methods are potentially useful and should be further developed and standardized.

## What Is Infodemiology?

The term infodemiology is a portmanteau of information and epidemiology. Epidemiology—the science of distribution and determinants of disease in populations—provides researchers, public health professionals, and policy makers with the tools and the data to influence public health and policy decisions. Unfortunately, with traditional epidemiological data collection methods, such as population health surveys, cohort studies, registries etc, it often takes years or decades to inform policy makers about the impact of public health policy decisions on public health. Also, early detection methods of outbreaks or other health conditions are often based on clinical data, and there is no “real-time” data on preclinical events and behavior patterns in a population.

*Infodemiology can be defined as the science of distribution and determinants of information in an electronic medium, specifically the Internet, or in a population, with the ultimate aim to inform public health and public policy*.

Potential infodemiology indicators and metrics include automatically aggregated and analyzed data on the prevalence and patterns of information on websites and social media; metrics on the “chatter” in discussion groups, blogs, and microblogs (eg, Twitter); and activities on search engines, etc.

Changes in information and communication patterns on the Internet can be an (early) “symptom” of changes in population health [1,2,4-6]. Reversely, in other situations, changes in information and communication patterns can have a negative or positive impact on population health as, for example, in the case of an “outbreak” of misinformation [9] or a public health campaign. Regardless of the direction of the arrow of causation, infodemiology is rooted in the idea that—at least for some areas and applications—there is a relationship between population health on one hand, and information and communication patterns in electronic media on the other, and if it were possible to develop robust metrics or “infodemiology indicators” which reflected these information and communication patterns in real-time, then all kinds of useful public health applications could be developed.

Thus, one important goal of infodemiology research is to develop, collect, and evaluate metrics and indicators for information and communication patterns that have some relationship to epidemiological data, or are otherwise useful for public health and policy making.

The term infodemiology was initially used to suggest development of measures for what is being published on the Internet (what is now called “supply-based infodemiology”) [9]. Much of the discussion in the late ’90s about the quality of health information on the Internet centered around the concern that low quality information on the Internet could be detrimental to public health [10], and it was in this context that the term was coined. For example, it is reasonable to assume that online campaigns by anti-vaccination groups have a real impact on vaccination rates and thus on health status. While we can measure the relationship between vaccination rates and health status, no sound methodologies exist to identify and track (automatically) the “prevalence” of information that leads to lower vaccination rates.

While "infodemiology" was first used in the context of analyzing the “supply side” (what is being published on the Web) [9], the scope of infodemiology now also includes “demand-based” infodemiology (ie, analyzing what people need and monitoring their health information seeking behavior) [1], because similar methods are employed.

Regardless of the source of information, infodemiology requires a set of novel methods for consumer and public health informatics to measure the epidemiology of information, describing and analyzing health information and communication patterns in electronic media (eg, on the Web). While it is conceivable that infodemiology metrics can also be obtained in the "offline-world", the focus on electronic media has a practical reason: Once information is available in electronic form, it can be automatically collected and analyzed. Supply- and demand-based infodemiology methods are similar in that they employ similar workflows and face similar problems: Selecting and filtering information (“concepts of interest”) from a large textual dataset, attempts to “understand” the information semantically (natural language processing), geocoding the information, and employing basic descriptive and analytical statistical methods, or more advanced temporospatial statistical methods to detect trends and clusters.

Using infodemiology data for surveillance purposes has been called “infoveillance” [8]. Infoveillance is important for both the supply and demand sides. Public health professionals want to know, for example, if there is a surge of misinformation on the Internet on vaccination, so that public health campaigns and “health marketing” efforts can effectively counterbalance the misinformation. Public health professionals also need to know about surges in information demand, be it to address “epidemics of fear” [3] by supplying the public with appropriate information, or to detect real disease outbreaks for which spikes in Internet searches or chatter in newsgroups and postings on microblogs (Twitter etc) may be an early predictor. Information on behavior change is itself an important intervention in the case of an outbreak, and tracking how effectively information is disseminated during a pandemic is another potential application.

In what follows, I will discuss supply-based indicators, demand-based indicators, and data sources in more detail. Together they form the vision for infodemiology, and also provide a blueprint for the Infovigil system.

## Supply-Based Infodemiology Methods and Applications

Imagine a system which continuously monitors Internet postings (be they on websites, blogs, microblogs, including Twitter, social media, discussion board postings, or other publicly available sources), employing natural-language processing and other methods to classify the postings by topic and obtaining indicators on changes over time. We call such metrics *supply*-based infodemiology indicators.

### Information (Concept) Prevalence

The most basic infodemiologic supply indicators are *information prevalence* and *information occurrence ratios* (or, perhaps more precisely, *concept prevalence* and *concept occurrence ratios*), measuring the absolute or relative number of occurrences of a certain keyword or concept in a pool of information. Note that we are talking about “keywords” if we simply look for the occurrence of certain terms, and "concepts" if we try to “understand” meaning, at a minimum combining multiple keywords to take into account synonyms.

The “pool of information” can be a set of documents, postings, status lines (Twitter, Facebook), a collection of Web pages, or websites. For example, we could, automatically, obtain estimates of the (absolute) number of Internet postings about a certain topic identified by a set of keywords. We call these kinds of data *information prevalence.* To be more specific on how we obtained the prevalence we could also talk about *keyword prevalence* or *concept prevalence*.

*Information prevalence* data are particularly useful if we track them longitudinally (ie, track how the number of Internet postings on a given health-specific topic changes over time), as we would, for example, to see changes in relation to certain external events, such as a media campaign or a disease outbreak.

A crude method to obtain these *prevalence* indicators is to enter a search term (with a Boolean OR to include synonyms) into a search engine, which provides an absolute number of occurrences over time (see however the caveat on the reliability of search engines below). An occurrence can (depending on the search engine) be either the number of documents containing the search term at least once, or can be the number of term occurrences in the entire database (the unit does not really matter for our purpose, as long as we use the same method consistently). More advanced methods would also take into account synonyms and do a semantic search (ie, tracking concepts as opposed to keywords), and/or filter the searches to focus on specific geographical regions (for example countries).

**Figure 1:**
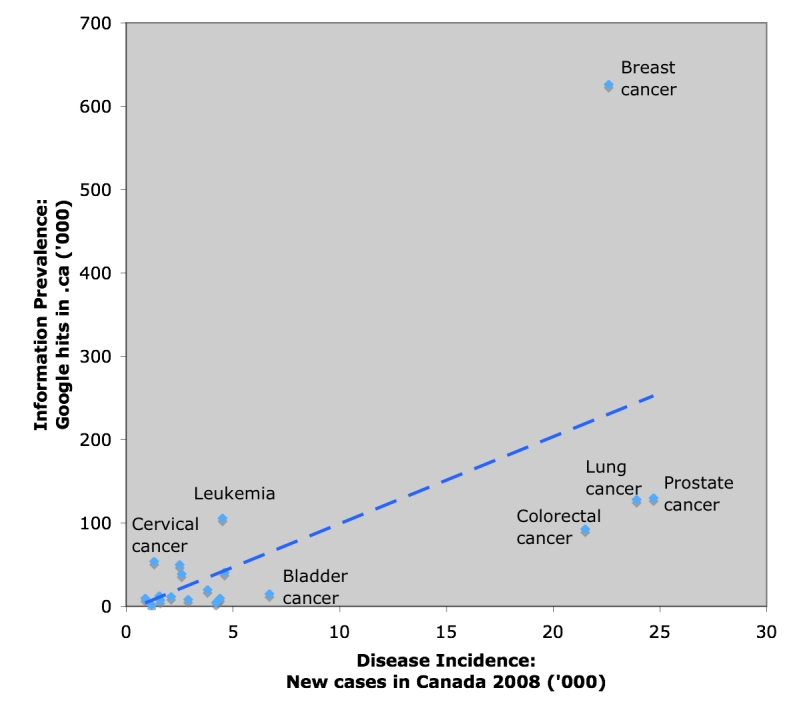
Information prevalence versus disease incidence scatterplot (Eysenbach, in preparation)

Figure 1 illustrates the information prevalence of various cancers in the Canadian top-level domain (.ca) plotted against actual disease incidence (a cautionary note: these data on information prevalence are based on crude Google hits rather than semantic analyses). Such *information prevalence versus disease incidence* scatterplots (or other comparators, for example information prevalence versus mortality) may be useful to illustrate to policy makers in which areas there may be an information deficit. From a public health perspective, diseases and conditions which have a high incidence and high disease burden (mortality rate or impact on quality of life), and which are preventable or for which screening tests exist, should enjoy better “coverage” in the media and on the Internet than those which are not. Thus, it is not expected or desirable that there is a strict correlation between cancer incidence and information prevalence. However, Figure 1 illustrates that—compared to other forms of cancer with similar disease burden—breast cancer is an extreme outlier, pointing to a larger health care disparity between, for example, breast and prostate cancers (which have similar incidence and mortality, yet receive different levels of attention and funding), which has been previously referred to as the “prostate cancer gap” [11]. Policy makers need to be aware of such inequalities and information gaps, and there is a role for supply-based infodemiology indicators, both for management of chronic diseases, as well as for management of public health emergencies. An "infodemiology dashboard" could be developed which displays some of these metrics to inform policy makers for which areas health marketing media campaigns are required.

### Information Incidence

As an analogy to the epidemiological terminology, we can also calculate information *incidence* rates, which determine the number of *new* information units created per unit of time. For example, comparing the incidence of Web pages which contain information about a certain topic, such as a new medical discovery, between countries, would provide interesting knowledge dissemination metrics.

**Figure 2:**
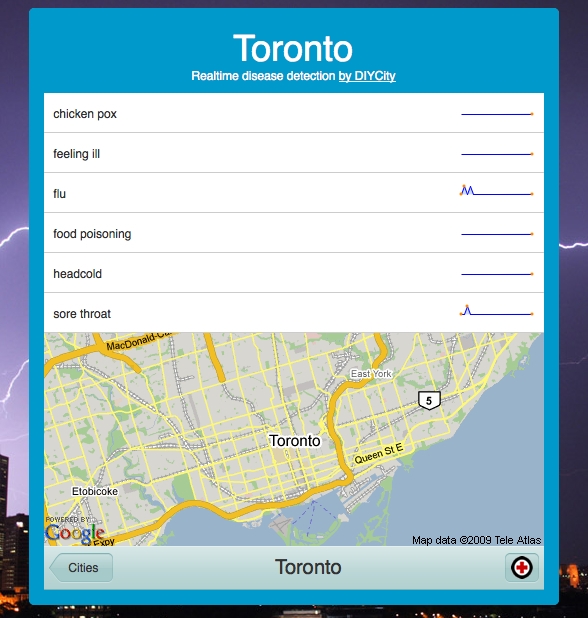
Information incidence (keyword occurrence) trends from Twitter status feeds ("tweets") (DIYCity/sickcity)

Information or concept incidence rates may also point to emerging public health threats. For example, the Infovigil project monitors Twitter microblogs for mentionings of public health relevant keywords and phrases, such as “I have fever”. Together with information on the location of the user, as well as automated conversations and directing users to surveys, these data can provide valuable information for public health agencies and the public alike. Figure 2 illustrates a very basic trend analysis of information incidence from Twitter feeds.

### Information (Concept) Occurrence Ratios

As the number of websites is constantly increasing, absolute figures on information prevalence are less meaningful than normalized indicators (ie, relative indicators such as rates and ratios). If the total number of “information units” in the “pool of information” is known, then the denominator used to normalize the absolute count could simply be the total number of information units. For example, if we know that the Web has a total of x Web pages in a given language at a given point in time, and y of these pages deal with cancer, then we can express the information prevalence as the proportion y/x. However, in the case of the Web, the denominator, which would be the total number of all indexed files and documents (including, for example, html, excel, and powerpoint files etc) in the specific language of the numerator keywords, is often hard to obtain or not known. While search engines such as Google may have data on the total number of indexed documents in a certain language, this information is usually proprietary and not accessible to researchers.

Thus, it is often easier is to express the information prevalence as a fraction of information units about a certain topic compared to a control keyword or concept. For example, if the number of Web resources mentioning “prostate cancer” is 21.6 million, compared to 214 million resources mentioning cancer, then the occurrence ratio of prostate cancer to cancer is 21.6:214 = 10%.

Studying occurrence ratios may provide fascinating insights into the linguistic and cultural differences of the use of words and concepts between countries, but it may also be a method to study inequalities and differences in access to health information. Table 1 illustrates differences between the information occurrence ratios for “cervical cancer” information versus “cancer” information in Canada, the United Kingdom, and Australia. However, these are crude analyses based on keyword on Google. A proper infodemiological investigation would attempt to “understand” semantically the content of Web pages.

Another important caveat is that many search engines do not give accurate or reliable hit counts. Not only provide different search engines different results, but even the same search engine queried multiple times during the same day may give different estimates. Systems like Infovigil collect this information from different search engines on different times during the day, and employs statistical methods to even out discrepancies. This also minimizes the potential bias that changes to the number of hits for certain keywords could be confounded by changes in search engine algorithms. Alternative methods exist that can bypass search engines altogether, for example random IP sampling or the random creation of domain names, but these methods have their own set of problems, such as triggering security alerts as they resemble hacking attempts.

**Table 1:** Information occurrence ratios for various concepts in English-speaking industrialized countries

	Google.com (all country domains)	Canada (.ca on Google.com)	United Kingdom (.uk on Google.com)	Australia (.au on Google.com)
**Hits (in Million)**				
"cervical cancer"	7.09	0.08	0.41	0.05
Cancer	227	3.95	8.77	3.03
Health	1190	49.7	57.4	67.8
Disease	226	3.67	4.84	3.05
Illness	68.6	1.57	2.8	2.02
Wellness	147	3.4	1.67	0.74
**Information Occurrence Ratios**				
cervical cancer/cancer	0.03	0.02	0.05	0.02
disease/health	0.19	0.07	0.08	0.04
disease/wellness	1.54	1.08	2.90	4.12
illness/health	0.06	0.03	0.05	0.03
illness/wellness ratio	0.47	0.46	1.68	2.73

### Concept Co-occurences

Looking for *co-occurrences* of different keywords or concepts (for example, a disease name and the name of a pharmaceutical substance) could provide *knowledge translation or innovation diffusion metrics*. For example, after publication of a trial confirming the effectiveness of a new drug in a medical journal, researchers could measure how long it takes for a new therapy to be acknowledged and taken up by the public, as reflected by the incidence of the disease term and the treatment concept occurring together. These indicators could in turn be useful to study different methods to accelerate knowledge translation (eg, publishing in open access journals, hosting workshops, holding press-conferences, and issuing press-releases, etc). Moreover, algorithms could be developed which monitor the medical, peer-reviewed literature, on the one hand, and the Internet, on the other, to collect and provide continuous real-time knowledge translation indicators.

While technically more challenging, it should also be possible to automatically identify and classify cases of misinformation or unbalanced information, tracking trends over time. For example, anti-vaccination websites use specific language, have specific attributes (eg, linking to other anti-vaccination sites), and cite a specific subset of the medical literature to provide a one-sided, biased view of the medical evidence [12]. A generic algorithm to obtain a measure for bias would, for example, be to compare the reference list of a systematic review to the references cited on a given website, which would enable researchers to quantify the direction and degree of content biases.

Once this information on the incidence of bias in a given field is collected in a longitudinal fashion, the effectiveness of public health and health marketing programs becomes measurable. For example, a media campaign addressing myths surrounding vaccination should lead to a change in the ratio of anti-vaccination postings to pro-vaccination statements, which in turn may be a predictor for changes in actual vaccination rates.

A final application area to be mentioned here is *policy implementation and evaluation*. As in the management adage which says, “one cannot manage what cannot be measured”, the case for gathering infodemiology data can be predicated on measuring the progress towards *policy objectives*, for example policies which address health information and health communication specifically related to the quality of information for the public. For example, the US public health policy document, “Healthy People 2010” [13] contained “[Increase of] quality of Internet health information sources” as an explicit policy objective (Objective 11-4). Other policy objectives (not from this document) may, for example, stipulate an increase of information written on a specific reading level, an increase of culturally sensitive health information for certain population groups or in certain languages (eg, minority languages). In most of these cases, it is conceivable that infodemiology methods could be developed and used to obtain and track indicators that would measure the progress towards such policy goals.

### Identifying and Aggregating Public-Health Relevant Information from Secondary Sources

Another class of “supply-side” based applications, for example the Global Public Health Intelligence Network (GPHIN), the HealthMap System, and the EpiSPIDER Project, analyze selected secondary data sources, such as news reports and expert newsletters (ProMED mail), and aggregate public-health relevant information, in particular about infectious disease outbreaks [14]. These systems can be seen as tools for Open Source Intelligence (OSINT) collection. OSINT is intelligence that is “produced from publicly available information that is collected, exploited, and disseminated in a timely manner to an appropriate audience for the purpose of addressing a specific intelligence requirement” (Sec. 931 of Public Law 109-163, National Defense Authorization Act for Fiscal Year 2006). (Note that in this context “open source” refers to publicly available information, not to open source software.)

These systems usually use a more selective approach in terms of choosing high-quality, expert-curated secondary data sources, as opposed to systems such as the Infovigil system, which attempt to harness the “collective intelligence” of people on the Internet by analyzing noisier "primary data" on information supply and demand (eg, Twitter feeds or search and navigation behavior).

### Identifying and Aggregating Public-Health Relevant Information on Social Networks

A final category of systems which could be developed would be systems which analyze and extract information from the Internet about the structure of social networks. For certain public health situations, especially in the case of an outbreak, but also for health marketing campaigns, it is advantageous to gather intelligence about the relationship between people. For example, it is conceivable that information on who knows whom from the friends-list on Facebook may help to contain the spread of an infectious agent if public health professionals have ready access to this information. Obviously, "knowing" somebody, communicating with someone, or being a "friend" with somebody on Facebook does not necessarily mean that these people have physical contact, hence, more advanced methods than just extracting the ""friends-list" from Facebook are required in order to be of use for public health.

## Demand and Navigation Behavior-Based Infodemiology Methods and Applications

With demand-based infodemiology indicators we usually refer to data generated from the search and “click” (ie, navigation) behavior of people.

Potential data sources include data from search engines (something only the search engines themselves have access to), as well as search and navigation data from Website logs (individual sites or pooled from different sites), although the latter is heavily confounded by the content of the website and thus more difficult to analyze. A final possibility is to develop a browser-plugin or desktop software that—with a user’s explicit consent—transmits anonymized search and possibly navigation behavior to the Infovigil data centre.

Query log data from search engines allow valuable insights into information needs and human behavior. Typical query log data contain a unique user identifier (eg, a random number, which is set as a cookie in the user’s client browser, allowing it to associate searches which come from the same user) and/or an IP address for the user, query string, query time, and the click URL. The user identifier and the IP address are privacy sensitive information. It is possible to reconstruct the identity of a user from this information if either data is left in the logfiles [15]. However, even search data that has been stripped of this information can be useful enough to identify trends [1].

Examples of *demand prevalence indicators* which can be construed from these data are the number of searches on a specific topic coming from a specific region, or the number of clicks on websites with a specific topic. While search data alone are sometimes ambiguous and often do not allow inference with the intention of the user (somebody looking for the keyword “cold” doesn’t necessarily have cold symptoms), search data analyzed in conjunction with click data are much more meaningful (for example, somebody looking for the keyword “cold” and then clicking on a link that says something like “Click here for comprehensive information on what to do if you have cold symptoms”, or just clicking on a medical website providing influenza information, provides richer and more specific information on the presumed intention of the user). It has also been shown that click data (on an influenza-specific ad) are more predictive for influenza than search data [1].

Search engine data mining methods exist that can use and cluster query and click data so that meaningful inferences can be made on the presumed intention of the user [16].

**Figure 3:**
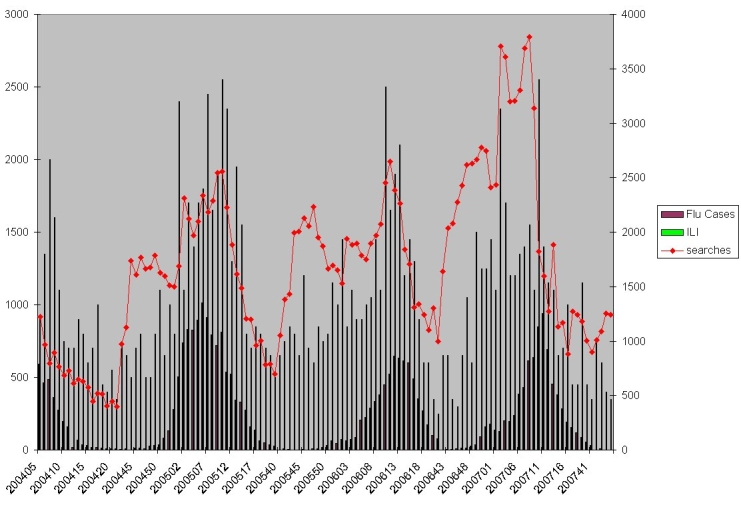
Relationship between information demand (searches on Google), flu cases, and influenza-like illnesses reported by sentinel physicians. Partial data from a five-year prospective study harvesting search and click data from Google 2004-2007 (Eysenbach 2007, presented at AMIA Annual Fall Symposium, Chicago 2007, method as described in [1])

## Active Methods Involving Consumers

The infodemiology and infoveillance methods outlined above can be referred to as passive methods, as they try to analyze and recognize trends on the Internet automatically and passively, without actively involving users. However, because the Internet is an interactive medium, there is also the potential to seamlessly collect even richer data from people, or to direct them to interventions. In the field of syndromic surveillance this represents nothing less than a paradigm shift, as traditional surveillance efforts, which are based, for example, on monitoring emergency room admissions or over-the-counter drug sales, happen without consumers even noticing it or being able to provide input. In contrast, using infoveillance methods, consumers can be directed to provide additional information. For example, when tracking search data for influenza specific keywords using the Google Ad method [1], it is possible to trigger an ad which leads to a quick online survey soliciting additional information from consumers. Similarly, postings in newsgroups or status updates on Twitter could trigger an automatic reply from an “infovigil robot” directing them to surveys or an intervention. Websites like whoissick.org or sicklike.me, which ask users to enter their symptoms, demonstrate that consumers are willing to actively participate in surveillance efforts by volunteering additional information.

## Bringing it all Together: Relationship Between Health Information Supply, Demand, and Population Health

To date, only a few pioneering studies have explored the relationships between information supply and/or demand, on one hand, and population health, on the other hand, and have experimented with infodemiology metrics. As argued above, the development and standardization of these metrics is an emerging area of research, and, as shown above, the application areas of infodemiology metrics range from early disease detection, to prevention and chronic disease management on a population level, to policy development and implementation, and knowledge translation research. Bringing demand- and supply-indicators together could allow for fascinating insights into the dynamics and interactions between information provision and information seeking on a population level.

A final perspective comes from the behavioral sciences. Analyzing how people search and navigate the Internet for health-related information, as well as how they communicate and share this information, can provide valuable insights to health-related behavior of populations, including for example the level of understanding of health issues, knowledge about health-related conditions, and so on. Such information is traditionally collected through large and expensive population surveys such as the National Cancer Institute's “Health Information National Trends Survey” (HINTS), which ask participants to recall their behavior. While such surveys remain invaluable, infodemiology methods and metrics may complement these data by providing direct and honest data on health information seeking behavior, free from recall or social desirability biases, conducted in real time, and executed relatively cheaply.

This fits into the broader vision of “populomics”, a term used by Gibbons to propose the gathering of “population level data to enable the development of ‘community (population) arrays’ or community-wide risk profiles” [17].

In 2006, the US National Cancer Institute and the Office of Behavioral and Social Sciences started to push for the concept of “populomics” to take its place alongside “genomics” and “proteomics”, arguing that “it is crucial for national planning and team science to include common data elements from the behavioral sciences into national assessments of population health”. The NCI further argued that “behavioral measures are needed in the health care environment [...] and in public health planning, in which national indices of progress on behavioral measures could guide policy and communication planning” [18].

Infodemiology metrics which reflect the behavior of people on the Internet, including their health seeking behavior or their behavior change attempts, such as smoking cessation, physical activity, dietary changes, use of sunscreen, and reduction in alcohol consumption, evidenced by search and publication behavior, could represent novel and valuable measures for this purpose.

## Conclusion

Infodemiology is an emerging discipline within public health informatics which characterizes a sign of our times: That it is not so much the availability of information that challenges us, but its aggregation and analysis. The field is highly interdisciplinary and requires the collaboration of information scientists, computer scientists, epidemiologists, medical experts, public health informatics experts, behavioral scientists, and statisticians. In fact, metrics and methods developed in other disciplines (infometrics, webometrics, and in the “open source intelligence” community) may have direct applications in infodemiology. Research partnerships with the private sector, for example search engines, is required. On the other hand, infodemiology also highlights threats to privacy and raises novel issues around informed consent, due to aggregation and analysis of openly accessible information about people on a large scale.

These challenges notwithstanding, the multitude of potential applications and benefits for society justify investments in infrastructure and research, and it is not least the peer-reviewers for funding agencies, philanthropical organizations, and medical journals which should keep an open mind to this novel and unconventional set of methods.
